# Neurophysiological indicators of internal attention: An electroencephalography–eye‐tracking coregistration study

**DOI:** 10.1002/brb3.1790

**Published:** 2020-08-20

**Authors:** Simon Majed Ceh, Sonja Annerer‐Walcher, Christof Körner, Christian Rominger, Silvia Erika Kober, Andreas Fink, Mathias Benedek

**Affiliations:** ^1^ Institute of Psychology University of Graz Graz Austria

**Keywords:** divergent thinking, electroencephalography, eye tracking, internal attention

## Abstract

**Introduction:**

Many goal‐directed and spontaneous everyday activities (e.g., planning, mind wandering) rely on an internal focus of attention. Internally directed cognition (IDC) was shown to differ from externally directed cognition in a range of neurophysiological indicators such as electroencephalogram (EEG) alpha activity and eye behavior.

**Methods:**

In this EEG–eye‐tracking coregistration study, we investigated effects of attention direction on EEG alpha activity and various relevant eye parameters. We used an established paradigm to manipulate internal attention demands in the visual domain within tasks by means of conditional stimulus masking.

**Results:**

Consistent with previous research, IDC involved relatively higher EEG alpha activity (lower alpha desynchronization) at posterior cortical sites. Moreover, IDC was characterized by greater pupil diameter (PD), fewer microsaccades, fixations, and saccades. These findings show that internal versus external cognition is associated with robust differences in several indicators at the neural and perceptual level. In a second line of analysis, we explored the intrinsic temporal covariation between EEG alpha activity and eye parameters during rest. This analysis revealed a positive correlation of EEG alpha power with PD especially in bilateral parieto‐occipital regions.

**Conclusion:**

Together, these findings suggest that EEG alpha activity and PD represent time‐sensitive indicators of internal attention demands, which may be involved in a neurophysiological gating mechanism serving to shield internal cognition from irrelevant sensory information.

## Graphical abstract

We report findings from the first electroencephalography (EEG)–eye‐tracking co‐registration study investigating effects of attention direction on EEG alpha activity and various relevant eye parameters. Our findings indicate that EEG alpha activity and PD can be viewed as time‐sensitive indicators of internal attention focus that are involved in a neurophysiological mechanism to gate task‐irrelevant sensory information in states of high memory load.

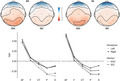

## 1 INTRODUCTION

We are often looking, but not always seeing, as we spend a considerable amount of time focusing our attention on internal processes (Killingsworth & Gilbert, 2010; Song & Wang, 2012). The term internally directed cognition (IDC; Chun, Golomb, & Turk‐Browne, 2011) refers to the devotion of attention to internal information and encompasses a wide range of mental activities such as imagination (Abraham, 2020), planning (Spreng, Stevens, Chamberlain, Gilmore, & Schacter, 2010), thinking about the past or the future (Baird, Smallwood, & Schooler, 2011), and more spontaneous forms such as mind wandering (Christoff, Irving, Fox, Spreng, & Andrews‐Hanna, 2016; Smallwood & Schooler, 2006). In contrast, attending to external information (e.g., during reading) is referred to as externally directed cognition (EDC). Importantly, we are limited in our ability to process information (Desimone & Duncan, 1995) and, therefore, IDC and EDC are considered to be competing states (Chun et al., 2011), sharing a common pool of resources (Verschooren, Schindler, De Raedt, & Pourtois, 2019). IDC was shown to differ from EDC in a range of neurophysiological indicators such as electroencephalography (EEG) alpha activity (Benedek, Bergner, Könen, Fink, & Neubauer, 2011; Cooper, Croft, Dominey, Burgess, & Gruzelier, 2003) and eye behavior (Smallwood et al., 2011; Walcher, Körner, & Benedek, 2017). In this study, we sought to (a) replicate the neurophysiological signature of IDC regarding EEG activity and eye behavior in a coregistration setting, employing the same manipulation of visual attention as in previous studies from our laboratory, and (b) test whether brain activation and eye behavior are directly related in terms of temporal covariation, potentially indicating that EEG alpha and specific eye behaviors are associated with a common gating mechanism in support of sustained internal attention.

### 1.1 EEG alpha activity and IDC

EEG alpha activity is the dominant rhythmic activity in the waking human brain (Klimesch, 1999) and is known to be sensitive to cognitive demands as evidenced by task‐related power (TRP) changes. Event‐related synchronization (ERS) reflects TRP increases, whereas event‐related desynchronization (ERD) reflects TRP decreases (Klimesch, Sauseng, & Hanslmayr, 2007). Traditionally, EEG alpha was assumed to be indicative of *cortical idling* (Pfurtscheller, Stancák, & Neuper, 1996), with higher alpha indicating states of cortical inactivity. This assumption was inferred from the observation that EEG alpha was particularly high during rest (e.g., eyes closed) and reduced when participants were engaged in cognitive tasks (Von Stein & Sarnthein, 2000). However, several studies also reported task‐related alpha increases for specific cognitive activities such as memory maintenance (Jensen, Gelfand, Kounios, & Lisman, 2002; Klimesch, Doppelmayr, Schwaiger, Auinger, & Winkler, 1999) and creative thinking (Agnoli, Zanon, Mastria, Avenanti, & Corazza, 2020; Stevens & Zabelina, 2019). Further lines of research suggested that alpha activity is increased for tasks that require internally directed attention (Ray & Cole, 1985), with evidence ranging across the visual, haptic, and acoustic sensory domain (Cooper et al., 2003).

Two studies from our laboratory have tested the role of attention direction in the context of creative thinking. In Benedek et al. (2011), we manipulated creative task demands using a convergent thinking task (anagram generation; i.e., less creative) and divergent thinking task (original sentence generation; i.e., more creative), and we independently manipulated attention demands using conditional stimulus masking that enforced participants to perform tasks mentally in half of the trials. Task‐related alpha activity was found to increase especially when tasks were performed under higher internal attention demands. In a second study (Benedek, Schickel, Jauk, Fink, & Neubauer, 2014), we explored whether alpha activity is increased during divergent thinking when the task is intrinsically independent from sensory processing (as most imagination tasks). To this end, we compared two divergent thinking tasks that differed in sensory intake demands: a sentence generation task, which relied on letter‐based processing and thus benefits from stimulus processing, and an alternate uses task, which relied on concept‐based processing and thus is largely unrelated to sensory processing. Indeed, the sensory‐independent alternate uses task involved much higher alpha activity compared to the sentence generation task, whereas alpha activity was increased during sentence generation only in the internal attention condition (masked stimuli). Other studies have also reported increased EEG alpha activity right before solving a creative problem with insight, which was seen as a “mental blink” to focus attention internally in order to evaluate the solution that just came to mind (Jung‐Beeman et al., 2004). Taken together, there is substantial evidence that alpha activity increases as a function of internal attention demands and plays a role in the inhibition of task‐irrelevant (sensory) processes to shield ongoing thought from distraction (Händel, Haarmeier, & Jensen, 2011; Rihs, Michel, & Thut, 2007; for reviews, see Benedek, 2018; Jensen & Mazaheri, 2010; Klimesch, 2012; Palva & Palva, 2007; Zabelina, 2018). It should be noted, however, that alpha activity is not necessarily a uniform phenomenon but may have different functions in sustained attention (Clayton, Yeung, & Kadosh, 2015, 2018), which may explain why relationships vary across topographic regions (e.g., Mo, Schroeder, & Ding, 2011).

### 1.2 Ocular mechanisms related to IDC

Internally directed cognition has also been linked to specific eye behavior changes, which may result from different attention‐related mechanisms. One ocular mechanism associated with IDC is *visual disengagement*, which refers to a reduced preparedness to detect and process visual information. For example, gaze aversion describes the phenomenon that people tend to avert their gaze during demanding cognitive tasks. Doing so may help to save cognitive resources by avoiding processing of irrelevant sensory information (e.g., during face‐to‐face conversation; Doherty‐Sneddon & Phelps, 2005). In fact, internal processes like mental arithmetic benefit from gaze aversion, especially when visual saliency is high (Abeles & Yuval‐Greenberg, 2017). Similarly, gaze aversion has been shown to become more frequent as tasks get more difficult (Doherty‐Sneddon, Bruce, Bonner, Longbotham, & Doyle, 2002). An even more apparent way of reducing visual input is eye closure. Closing our eyes enhances performance in IDC tasks, as evidenced by studies regarding convergent and divergent creativity (Ritter, Abbing, & Van Schie, 2018) and insight problem solving (Salvi, Bricolo, Franconeri, Kounios, & Beeman, 2015). In addition, microsaccadic activity, a mechanism to counteract perceptual fading (Martinez‐Conde, Macknik, Troncoso, & Dyar, 2006; Martinez‐Conde, Otero‐Millan, & Macknik, 2013), has been shown to decrease during IDC (Benedek, Stoiser, Walcher, & Körner, 2017; Gao, Yan, & Sun, 2015). Similarly, eye vergence is linked to shifts in visuospatial attention (Solé Puig, Puigcerver, Aznar‐Casanova, & Supèr, 2013). Vergence movements refer to the rotation of the eyes in opposite directions to obtain single binocular vision. First evidence suggests that the angle of eye vergence (AoEV; Solé Puig, Puigcerver, et al., 2013) may serve as an indicator of internal attention (Benedek et al., 2017; Huang, Li, Ngai, Leon, & Bulling, 2019). Studies from our laboratory have further connected goal‐directed IDC with increases in blink rate (Walcher et al., 2017) and blink duration (Benedek et al., 2017), both contributing to reduced sensory input in support of sustained internal attention.

A second ocular mechanism related to IDC is *perceptual decoupling* (Smallwood & Schooler, 2006). It describes the capacity for our minds to flexibly disengage attentional processes from sensory input (Smallwood & Schooler, 2015). Specifically, during IDC, eye behavior has been shown to become less guided by stimulus characteristics of the external visual environment. As an example, mind wandering has been linked to more spontaneous baseline pupillary activity, which was accompanied by more erroneous responses during a working memory task and higher baseline PD (Smallwood et al., 2011), the latter also being found in a different study (Franklin, Broadway, Mrazek, Smallwood, & Schooler, 2013). Other studies challenge these findings, showing decreased PD prior to off‐task episodes (Unsworth & Robison, 2016; Grandchamp, Braboszcz, & Delorme, 2014). Despite these inconsistencies that may in part be attributable to task characteristics (cf. Grandchamp et al., 2014), these studies suggest that eye behavior was no longer coupled to task‐relevant sensory input. Also, during mindless reading, fixations are less affected by text characteristics (Reichle, Reineberg, & Schooler, 2010), again pointing toward a decoupling from external information.

Yet another ocular mechanism related to IDC is *internal coupling*. When we engage in internal processes, eye behavior may not only decouple from the external environment, but even couple to the internal processes. This is evidenced by changes in pupil diameter due to imagined luminance changes (Laeng & Sulutvedt, 2014), as well as by changes in pupil diameter and vergence eye movements in response to the size and distance of imagined objects (Sulutvedt, Mannix, & Laeng, 2018). Taken together, these ocular mechanisms contribute to the eye behavior changes observed during IDC. Hence, for tasks with well‐controlled sensory demands, eye behavior can serve as physiological indicator of an internal versus external focus of attention.

### 1.3 Aims of this study

So far, it has been established that IDC is associated with EEG alpha increases (Benedek & Fink, 2019; Jensen & Mazaheri, 2010; Klimesch et al., 2007) as well as with specific eye behavior changes (e.g., Benedek et al., 2017; Salvi et al., 2015). Most evidence comes from studies that manipulated visual attention, some of these even used the same experimental design across separate EEG and eye‐tracking studies (cf. Jung‐Beeman et al., 2004 and Salvi et al., 2015, or Benedek et al., 2011 and Benedek et al., 2017). Importantly, EEG alpha activity and eye behavior may play a role in supporting sustained internal attention: EEG alpha is thought to inhibit task‐irrelevant sensory processing (Benedek, 2018; Jensen & Mazaheri, 2010; Klimesch, 2012), and eye behavior can contribute to reduced sensory uptake (Huang et al., 2019; Walcher et al., 2017). This raises the question whether EEG alpha and specific eye behaviors are associated with a common neurophysiological gating mechanism facilitating IDC (cf. Chatham & Badre, 2015).

Here, we examined EEG alpha activity and eye behavior in the contexts of both experimentally induced and spontaneous variations of internal attention. Specifically, we tested (a) whether EEG alpha and eye behavior differences between IDC and EDC can be robustly observed within the same study using a well‐established experimental manipulation of internal attention demands and (b) whether EEG alpha is directly related to specific eye behavior changes in terms of temporal covariation of EEG alpha and eye behaviors during rest. A correlation of EEG alpha activity and eye behavior across conditions and time would corroborate their role as indicators of transient changes in attention focus and add weight to the assumption that oscillatory activity in the alpha band is involved in modulating perception (Jensen, Bonnefond, & VanRullen, 2012).

## 2 METHODS

Materials, data, and analysis scripts are provided on the Open Science Framework (OSF, https://doi.org/10.17605/OSF.IO/5U6R9).

### 2.1 Participants

Fifty‐one students participated in this study. Fifteen participants were excluded from further analyses, because of excessive missing data (*n* = 14; exclusion criteria are presented below) or due to technical problems (*n* = 1) during data acquisition. The final sample thus consisted of 36 subjects (24 female), with an average age of 24 years (*SD* = 2.72). All participants were right‐handed, had normal (*n* = 32) or corrected‐to‐normal (*n* = 4, soft contact lenses) vision, and reported neither medical or psychological disorders, nor problems regarding vision (e.g., cataract, strabismus). Eye sight was also tested using Landolt rings (Wesemann, 2002). They gave written informed consent prior to the start of the EEG and eye‐tracking study and were to choose between financial compensation and partial course credit. The procedure of this study was approved by the local ethics committee.

### 2.2 Apparatus

Participants were seated in a darkened and sound‐attenuated EEG cabin, and written informed consent was obtained. While participants read through the instructions of the experiment, electrodes were mounted and impedances checked. EEG was recorded with a BrainAmp amplifier (Brain Products GmbH) at 1,000 Hz using 19 active electrodes, positioned according to the 10‐20 system. The ground electrode was placed centrally on the forehead, and the reference electrode was placed at the left mastoid. For later rereferencing, another electrode was placed at the right mastoid. Moreover, three EOG (electrooculogram) electrodes were included, placed left and right of the eyes, and adjacent to the radix nasi. Impedances of the electrodes were typically kept < 30 kΩ (<10 kΩ for reference electrodes). Minor violations of these criteria were occasionally tolerated for single electrodes to ensure reasonable preparation time.

Binocular eye‐tracking data were acquired using the EyeLink 1000 Plus eye tracker (SR Research Ltd.) with a temporal resolution of 1,000 Hz. Participants sat at a distance of approx. 70.5 cm from the screen. Head movements were limited using a chin rest (we did not use a forehead rest to avoid possible pressure artifacts on frontal electrodes). Stimuli were presented on a 24” Samsung S24A450 Monitor (Samsung Electronics Co., Ltd.) run at 60 Hz with a native resolution of 1,900 × 1,200 pixels. The experiment was run at 1,280 × 1,024 pixels resolution. Stimuli were presented using the EyeLink Experiment Builder software (version 2.1.512; SR Research Ltd.). We conducted a 9‐point calibration and validation at the beginning of the experiment and drift correction before each trial. The latter was done to account for minor deviations from the intended sitting position over the time course of the experiment.

Proper synchronization of the data records was ensured by using TTL triggers that were simultaneously sent to both the eye‐tracking and EEG system. Synchronization was verified with the EYE‐EEG toolbox (Dimigen, Sommer, Hohlfeld, Jacobs, & Kliegl, 2011) for EEGLAB (v. 14.1.2; Delorme & Makeig, 2004).

### 2.3 Experimental design, tasks, and procedure

The experimental session included two parts. In the first part, EEG and eye tracking were recorded during a two‐minute resting period with open eyes during which participants were instructed to keep eyes open and directed toward a fixation cross. Such an extended resting state condition is commonly used to assess intrinsic neurophysiological activity (Raichle, 2015) and is associated with episodes of mind wandering (Smallwood & Schooler, 2015). Hence, this resting state assessment reflects spontaneous variations in attentional focus (external vs. internal), which enables the examination of the intrinsic covariation of attention‐related neurophysiological indicators.

In the second part, EEG and eye tracking were recorded during performance of two tasks where internal attention demands were experimentally manipulated within tasks. The experimental tasks and design were closely adapted from previous research (Benedek et al., 2011, 2016, 2017). Specifically, participants performed a convergent (anagram generation; AN) and a divergent thinking task (sentence generation; SG), under low and high internal attention demands (see below), resulting in a 2 × 2‐within‐subjects design. In each trial, a meaningful German four‐letter word was presented. In the anagram task, participants were required to rearrange the four letters, in order to build another meaningful four‐letter word (e.g., “ROBE” is transformed to “BORE”). In the SG task, participants were asked to generate a meaningful 4‐word sentence, using each letter as an initial letter of one word (e.g., “ROBE” is transformed to “Robert observes eye behavior”); each letter had to be used once, but not necessarily in the original order.

Each trial consisted of an initial *drift check*, which also ensured that participants fixated the center of the screen from the beginning, followed by a 5 s reference phase, during which participants were asked to look at a fixation cross. This reference phase would form a trial‐wise baseline for later computation of TRP changes. At the beginning of the activation phase (20 s), the stimulus was presented in black letters on gray background. In the internal condition (50% of trials), the stimulus was masked by replacing the four stimulus letters with “XXXX” after 500 ms, forcing participants to perform the task in their mind's eye after stimulus encoding for the remaining 19.5 s. In the external condition (other 50% of trials), the stimuli remained visible for the entire 20 s, thus allowing continuous sensory processing. After that timespan, the original stimulus reappeared in green color, signaling that the response should be vocalized (response phase; 6 s).

Participants were instructed that if they came up with a solution before the end of the task, they should keep thinking of further solutions (AN task) or more original solutions (SG task) until the beginning of the response phase. Up to three correct solutions were possible in the AN task, whereas in the SG task, the amount of possible responses was potentially unending. An item was correctly solved, when at least one of the possible anagram solutions had been found, or when a grammatically correct sentence with all four target letters had been produced.

The trials were organized into blocks of six items from the same task to reduce task switching costs. Each block started with a task cue announcing the relevant task (AN or SG). Within each block, three trials were realized in the internal and three in the external condition, in a randomized order. This resulted in a total of nine trials for each combination of task and condition. The trial order was identical for each participant; yet, participants were pseudo‐randomly assigned to an ABBAAB, or BAABBA sequence of task blocks (A = AN task, B = SG task), which ensured that all items were performed in both tasks for different participants. After completing the tasks, participants rated difficulties for each task in each condition on a 5‐point Likert scale ranging from 0 (very easy) to 4 (very difficult).

### 2.4 Data preprocessing and analysis

#### 2.4.1 EEG data

Electroencephalography data preprocessing was done with BrainVision Analyzer (v. 2.1, Brain Products GmbH). In a first step, we applied a notch filter (50 Hz) to make sure that the signal was not contaminated by power line frequency. Data were downsampled to 100 Hz and then rereferenced to the average of both reference electrodes. We visually inspected data and marked any noneye‐related artifacts (i.e., muscular activity and movement artifacts) to be excluded from further analysis. The coregistration design further enabled us to remove eye movement artifacts based on blink events and saccadic activity detected by the eye tracker. We conducted a side analysis to determine the effects of blinks, as classified by the eye tracker, on EEG alpha power (see Figure A in the Supporting Information). It revealed that blink‐related influences on EEG alpha power were apparent in a time window ranging from about 350 ms before detected blink onsets to about 200 ms after detected blink offsets. Therefore, this extended blink period was excluded from further analysis. To account for influences of saccades on EEG activity (Keren, Yuval‐Greenberg, & Deouell, 2010), we also removed intervals ranging from 20 ms before to 20 ms after each saccade. These steps helped to avoid that attention effects on EEG alpha activity are caused by condition‐related differences in blink frequency, blink duration or saccade count.

We computed continuous alpha band power (8.50–12.50 Hz), using the frequency extraction method implemented in BrainVision Analyzer based on complex demodulation. This frequency band captured peak alpha activity as evidenced by analyses of average frequency power (see Figure B in the Supporting Information). Task‐related alpha power (TRP) for a given electrode *i* was computed by subtracting the mean alpha power (POW) in the reference phase from the mean alpha power in the activation period of each trial according to the formula (cf. Benedek et al., 2011):

$${\text{TRP}}_{i}=\log\left(1+\text{mean}\left({\text{POW}}_{i},\text{activation}\right)\right)‐\log\left(1+\text{mean}\left({\text{POW}}_{i},\text{reference}\right)\right)$$

Negative values thus reflect task‐related alpha desynchronization, whereas positive values reflect alpha synchronization. The reference period consisted of a 5 s fixation cross prior to stimulus onset of which the first and last 500 ms were discarded. The activation interval ranged from 1.5 s after stimulus presentation (i.e., 1 s after conditional onset of stimulus masking) to the end of stimulus presentation 18.5 s later. For statistical analyses, the electrodes were topographically aggregated as follows: anteriofrontal (AF) left (FP1) and right (FP2), frontal (F) left (F3, F7) and right (F4, F8), centro‐temporal (CT) left (T7, C3) and right (T8, C4), parietal (P) left (P3, P7) and right (P4, P8), and occipital (O) left (O1) and right (O2). Midline electrodes were not included, as hemispheric differences were investigated.

#### 2.4.2 Eye‐tracking data

Data Viewer (SR Research Ltd.) was used to obtain relevant eye behavior data (i.e., pupil, blink, and gaze position data). Eye parameters were computed from this data using R scripts (www.r‐project.org) similar to previous research (cf. Benedek et al., 2017): For the calculation of pupil diameter (PD) and AoEV, the data were downsampled to 100 Hz by averaging across 10 data points (10 ms). PD was defined as the average across both eyes. After removing outliers (±3 SD) and visual inspection, we z‐scored PD data within subjects across the rest and performance periods, respectively. The AoEV was calculated following previous studies (Benedek et al., 2017; Walcher et al., 2017) using an established formula (Solé Puig, Zapata, Aznar‐Casanova, & Supèr, 2013) based on in the intersection point of eye vectors and using a mean interpupil distance of 60 mm. For each trial, valid PD and AoEV data were averaged across the activation interval, and variance was additionally computed as index of parameter variability (i.e., PD variance, and AoEV variance; Smallwood et al., 2011). Saccades, microsaccades, and their respective amplitude were determined using the Microsaccade Toolbox for R (Engbert, Sinn, Mergenthaler, & Trunkenbrod, 2015) using the same threshold settings as in previous studies (Benedek et al., 2017), with microsaccades being defined as saccades with an amplitude below 1° v.a. lasting for at least 6 ms and *λ* = 4. Blink count and blink duration were computed on the basis of the aforementioned output data from Data Viewer. We only considered binocular blinks as defined by the eye tracker's built‐in detection algorithm. Fixations were defined as intervals that did not involve blinks or saccades and their frequency and duration was computed.

Task‐based analyses of eye behavior focused on the 18.5 s activation interval starting 1.5 s after stimulus onset (just as for the EEG data), to account for possible influences related to stimulus presentation (cf. Nikolaev, Meghanathan, & Van Leeuwen, 2016). To ensure robust analyses, we only maintained trial and person data that met the following criteria: Only trials with correct responses, containing more than 500 ms artifact‐free data in the reference and 33% artifact‐free data in the activation periods (considering both EEG and eye‐tracking artifacts), were included in further analysis. Participants failing to show a minimum of 33% (=3/9) valid trials per task in each condition or showing less than 50% (=18/36) correct trials overall were excluded from further analysis. Bonferroni correction was applied to account for multiple comparisons (i.e., critical alpha was set to 0.00417).

### 2.5 EEG–eye‐tracking covariation analysis

**Figure 1:**
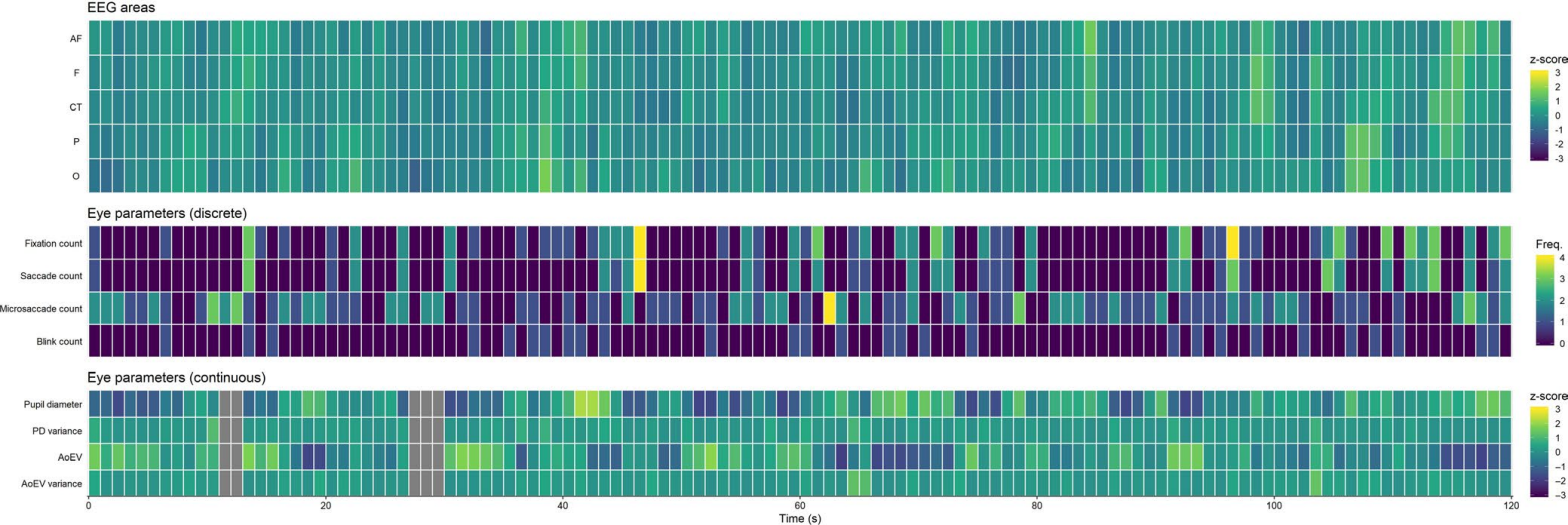
Exemplary segmented data of the 120 s resting state period of one participant, which forms the basis of the electroencephalography (EEG)—eye‐tracking covariation analysis. All EEG and eye‐tracking parameters were segmented to 1‐s segments represented by single tiles. Continuous parameters (EEG alpha, and PD, PD variance, AoEV, and AoEV variance) were z‐scaled; for discrete parameters, the frequency of occurrence within the time segment was counted. AF, anteriofrontal; AoEV, Angle of Eye Vergence; CT, centro‐temporal; F = frontal; O, occipital; P, parietal; PD, pupil diameter. Gray tiles mark missing data

The temporal correlation of EEG alpha power and eye behavior was analyzed for the 120 s rest period. We only included subjects for which at least 66% artifact‐free data existed, as this analysis relies on the continuity of the data. The resulting sample for the analysis of temporal correlation comprised 44 participants. The rest period was split into 120 1 s segments to ensure that all eye parameters (including discrete measures like blinks or saccades) can be related to EEG alpha power at a common time scale. For each segment, we computed mean scores for continuous parameters (e.g., PD) and frequencies for discrete parameters (e.g., blinks). Further deviations from data preprocessing as outlined for the task period involved the use of logarithmic EEG alpha power instead of TRP (no reference period) and z‐scoring of all continuous parameters (i.e., PD, PD variance, AoEV, AoEV variance & EEG channels) within subjects to increase their comparability. An example for the resulting data structure is presented in Figure 1.

For each participant, Pearson's correlations between EEG alpha activity and eye parameters were calculated over artifact‐free time periods (i.e., valid EEG and eye‐tracking data) for each EEG region, separately. A side analysis revealed no lateralization effects regarding the covariation of EEG and eye‐tracking data. Therefore, in our covariation analysis, we present EEG regions aggregated across both

hemispheres. Resulting correlations were Fisher's z‐transformed before being averaged across participants. Then, the average Fisher's z‐values were retransformed to correlation coefficients, and confidence intervals were computed to test whether mean correlations are different from 0. Bonferroni correction was applied to account for multiple comparison.

## 3 RESULTS

### 3.1 Behavioral results

Participants were able to solve 82.72% (*SE* = 1.30) of all trials. A 2 × 2 repeated measures ANOVA with regard to the within‐subject factors ATTENTION (IDC vs. EDC) and TASK (AN vs. SG) was conducted in order to further investigate task performance. Significant main effects were found for ATTENTION (*F*_1,35_ = 9.00, *p* = .005, $\eta_{p}^{2}$ = 0.20), showing that internal task performance was lower (*M* = 79.94%, *SE* = 1.94) compared to task performance when stimuli were continuously available (*M* = 85.49%, *SE* = 1.70) and TASK (*F*_1,35_ = 9.50, *p* = .004, $\eta_{p}^{2}$ = 0.21), indicating that the anagram task (*M* = 87.04%, *SE* = 1.54) was easier than the sentence generation task (*M* = 78.40%, *SE* = 1.99). There was no significant interaction between ATTENTION and TASK (*F*_1,35_ = 0.46, *p* = .50).

These effects were also reflected in subjective difficulty ratings. A repeated measures ANOVA revealed significant main effects for both ATTENTION (*F*_1,35_ = 79.29, *p* < .001, $\eta_{p}^{2}$ = 0.69) and TASK (*F*_1,35_ = 15.73, *p* < .001, $\eta_{p}^{2}$ = 0.31), but no significant interaction between ATTENTION and TASK (*F*_1,35_ = 0.66, *p* = .42). Internal task performance (*M* = 2.43, *SE* = 0.12) was rated more difficult than external task performance (*M* = 1.54, *SE* = 0.10), and the anagram task (*M* = 1.64, *SE* = 0.12) was found to be easier than the sentence generation task (*M* = 2.33, *SE* = 0.11). These results are consistent with previous studies using the same paradigm in showing that task performance in the mind's eye is more difficult due to the increased memory load (Benedek et al., 2011, 2017).

### 3.2 EEG results

**Figure 2:**
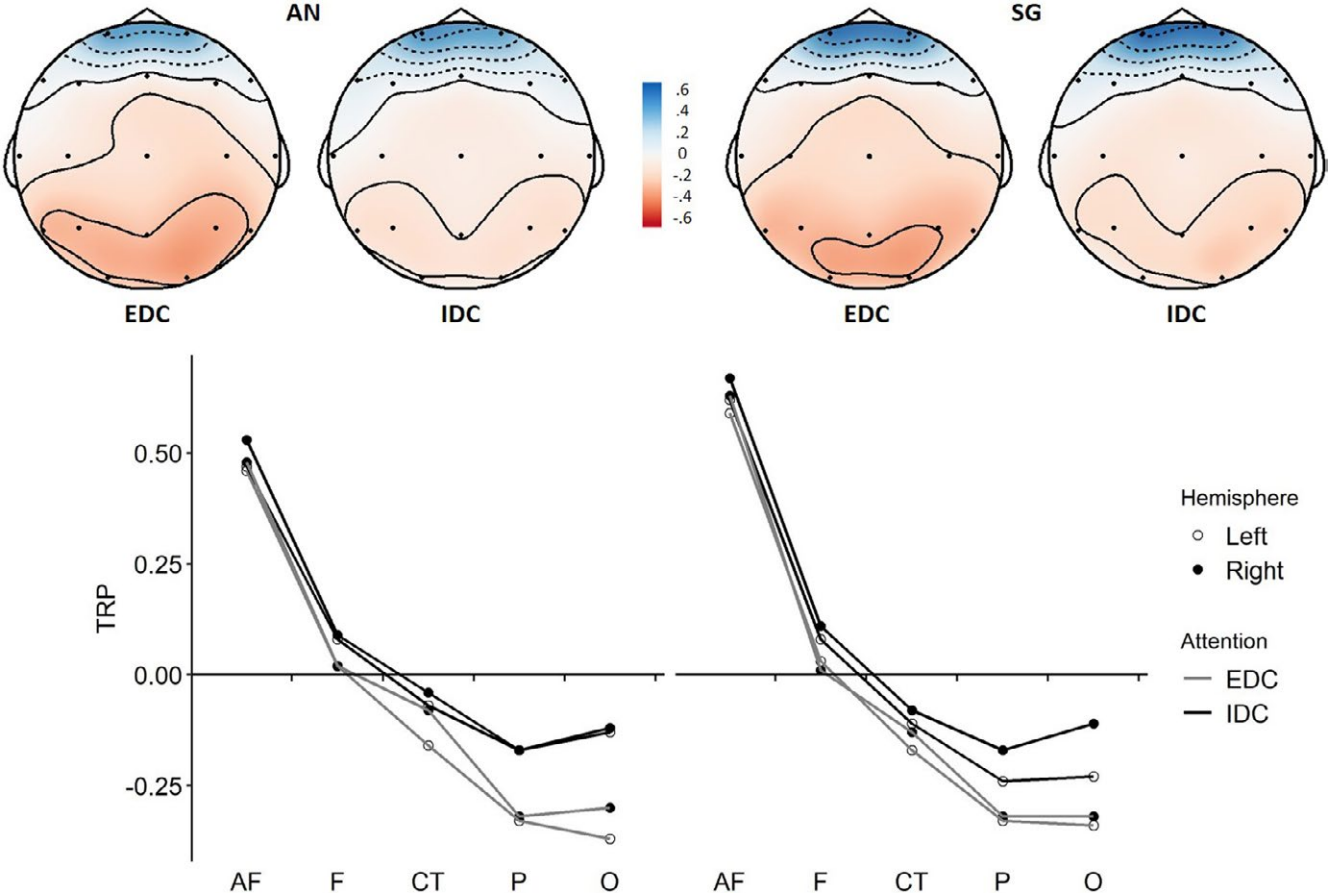
Task‐related power (TRP) changes in the alpha band (8.5–12.5 Hz) during anagram generation (AN) and sentence generation (SG) for experimental conditions of externally directed cognition (EDC) versus internally directed cognition (IDC). Positive TRP (cool colors) indicates task‐related alpha synchronization, and negative TRP (warm colors) indicates desynchronization (AF, anteriofrontal; CT, centro‐temporal; F = frontal; P, parietal; O, occipital). IDC resulted in relatively higher TRP in posterior brain regions for both tasks

The TRP changes in the alpha band were analyzed using repeated measures ANOVAs with within‐subject factors ATTENTION (IDC vs. EDC), TASK (AN vs. SG), HEMISPHERE (left vs. right), and AREA (anteriofrontal, frontal, centro‐temporal, parietal, and occipital). The 2 × 2 × 2× 5 ANOVA revealed a significant main effect ATTENTION (*F*_1,35_ = 14.62, *p* < .001, $\eta_{p}^{2}$ = 0.29), a significant main effect AREA (*F*_4,140_ = 44.57, *p* < .001, $\eta_{p}^{2}$ = 0.24), as well as a significant interaction ATTENTION × AREA (*F*_4,140_ = 6.31, *p* < .001, $\eta_{p}^{2}$ = 0.04), indicating that IDC led to smaller TRP decreases (i.e., relatively higher alpha activity) than EDC at parietal and occipital sites (lower desynchronization; see Figure 2; see also Figure D in the Supporting Information, which plots the time course of occipital TRP for EDC/IDC conditions: It shows that attention‐related differences in EEG alpha are observed early on but tend to become less pronounced as the task progresses). There was no main effect for TASK (*F*_1,35_ = 0.44, *p* = .512), but a significant effect of HEMISPHERE (*F*_1,35_ = 4.81, *p* = .035, $\eta_{p}^{2}$ = 0.12), indicating relatively higher TRP over the right hemisphere. We further observed a TASK × AREA interaction (*F*_4,140_ = 6.97, *p* < .001, $\eta_{p}^{2}$ = 0.05), suggesting that during anagram generation, there was relatively lower TRP in the anteriofrontal region compared to sentence generation. All other interaction effects were not significant (TASK × ATTENTION: *F*_1,35_ = 0.31, *p* = .582; TASK × HEMISPHERE: *F*_1,35_ = 0.46, *p* = .503; ATTENTION × HEMISPHERE *F*_1,35_ = 0.64, *p* = .429; HEMISPHERE × AREA: *F*_4,140_ = 0.86, *p* = .488; TASK × ATTENTION × HEMISPHERE: *F*_1,35_ = 2.80, *p* = .103; TASK × ATTENTION × AREA: *F*_4,140_ = 0.57, *p* = .685; TASK × HEMISPHERE × AREA: *F*_4,140_ = 0.55, *p* = .699; ATTENTION × HEMISPHERE × AREA: *F*_4,140_ = 0.95, *p* = .436; TASK × ATTENTION × HEMISPHERE × AREA: *F*_4,140_ = 1.99, *p* = .099). These results show that IDC conditions resulted in relatively higher EEG alpha activity in posterior brain regions across both tasks (see Figure 2; for full descriptive statistics, see Table 1 in Supporting Information).

To check whether the TRP difference between internal and external attention conditions resulted from alpha power differences in the reference or activation period, we computed additional ANOVAs for the logarithmic power of the reference and activation periods. Significant effects for ATTENTION (*F*_1,35_ = 32.55, *p* < .001, $\eta_{p}^{2}$ = 0.48) and ATTENTION × AREA (*F*_4,140_ = 14.79, *p* < .001, $\eta_{p}^{2}$ = 0.10) were found for alpha power in the activation period, whereas for the reference period, no significant attention effects were observed (ATTENTION: *F*_1,35_ = 0.60, *p* = .44; ATTENTION × AREA: *F*_4,140_ = 0.94, *p* = .44), suggesting that the observed attention effects on TRP are due to differences during the activation period rather than the reference period.

### 3.3 EYE‐tracking results

**Figure 3:**
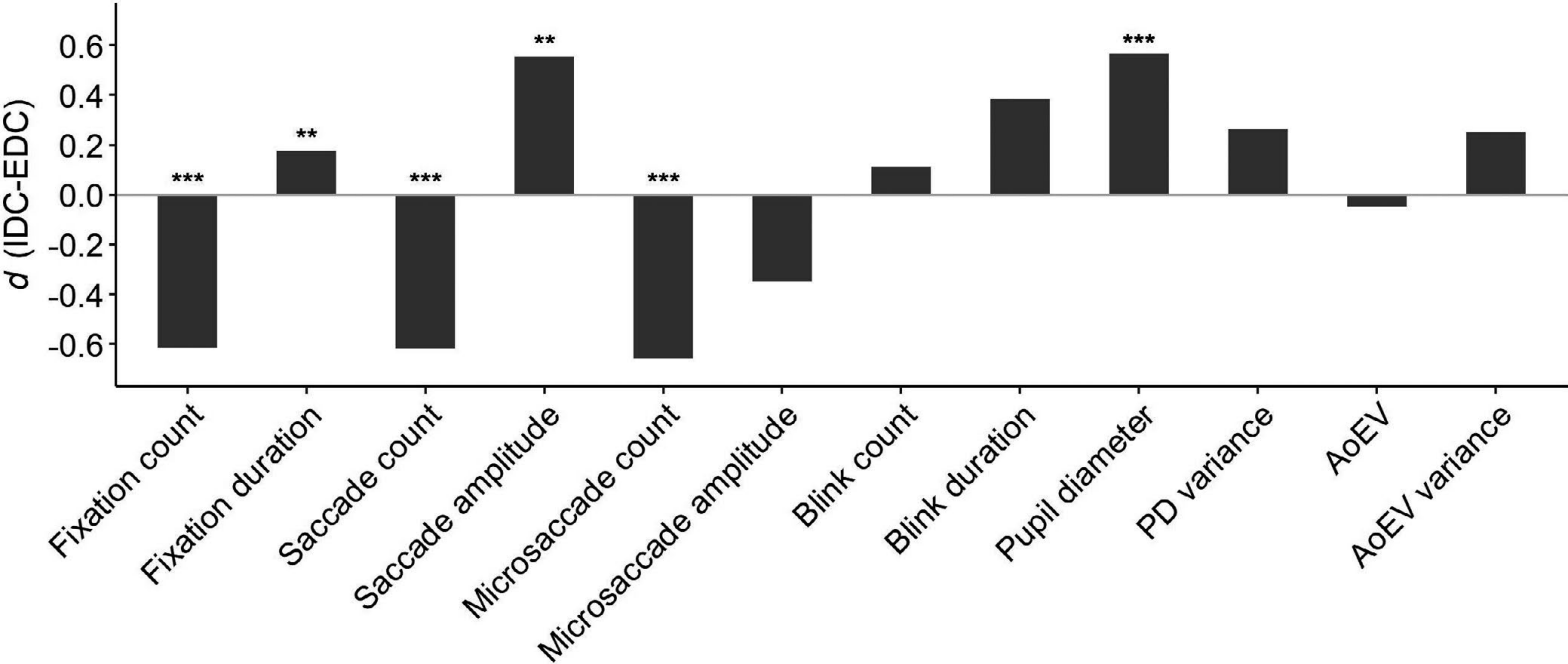
Effect sizes of oculometric differences between internally directed cognition (IDC) and externally directed cognition (EDC). AoEV, angle of eye vergence; PD, pupil diameter. ***p < .001, **p < .01

Attention effects (ATTENTION: IDC vs. EDC) and potential task effects (TASK: AN vs. SG) on available oculometric parameters were analyzed with 2 × 2 ANOVAs for each eye parameter. Looking at attention effects first (main effect ATTENTION), IDC involved lower fixation count (*F*_1,35_ = 20.10, *p* < .001, $\eta_{p}^{2}$ = 0.36), higher fixation duration (*F*_1,35_ = 10.64, *p* = .002, $\eta_{p}^{2}$ = 0.23), lower saccade count (*F*_1,35_ = 18.55, *p* < .001, $\eta_{p}^{2}$ = 0.35), higher saccade amplitude (*F*_1,34_ = 5.34, *p* = .003, $\eta_{p}^{2}$ = 0.14), lower microsaccade count (*F*_1,35_ = 97.77, *p* < .001, $\eta_{p}^{2}$ = 0.74), and higher PD (*F*_1,35_ = 32.17, *p* < .001, $\eta_{p}^{2}$ = 0.48). There were no significant attention effects on microsaccade amplitude (*F*_1,35_ = 5.47, *p* = .03), blink count (*F*_1,35_ = 5.73, *p* = .022), blink duration (*F*_1,35_ = 6.44, *p* = .016), AoEV (*F*_1,35_ = 1.56, *p* = .22), PD variance (*F*_1,35_ = 6.28, *p* = .017, $\eta_{p}^{2}$ = 0.12), nor AoEV variance (*F*_1,35_ = 3.73, *p* = .062) at the 0.00417 level. Effect sizes of all oculometric differences between IDC and EDC are displayed in Figure 3 (for full descriptive statistics, see Table 2 in the Supporting Information); Figure D in the Supporting Information, plots the time course of PD for EDC/IDC conditions: It shows that attention‐related differences in PD are observed consistently across the trial period but tend to become less pronounced as the task progresses.

These attention effects showed high consistency across both tasks, as no ATTENTION × TASK interactions were observed for fixation count (*F*_1,35_ = 4.18, *p* = .048), fixation duration (*F*_1,35_ = 0.43, *p* = .517), saccade count (*F*_1,35_ = 6.08, *p* = .019), saccade amplitude (*F*_1,34_ = 2.66, *p* = .112), microsaccade count (*F*_1,35_ = 8.58, = 0.006), microsaccade amplitude (*F*_1,35_ = 3.40, *p* = .080), blink count (*F*_1,35_ = 0.38, *p* = .540), blink duration (*F*_1,35_ = 1.60, *p* = .214), PD (*F*_1,35_ = 0.04, *p* = .847), PD variance (*F*_1,35_ = 3.50, *p* = .070), AoEV (*F*_1,35_ = 0.15, *p* = .701), nor AoEV variance (*F*_1,35_ = 0.98, *p* = .329).

A significant TASK effect was found for PD (*F*_1,35_ = 69.45, *p* < .001, $\eta_{p}^{2}$ = 0.66), showing that the SG task involved higher PD in both attention conditions. No significant task effects were observed for the other oculometric parameters at the 0.00417 level (Bonferroni corrected).

### 3.4 Temporal covariation between EEG alpha power and eye behavior

**Figure 4:**
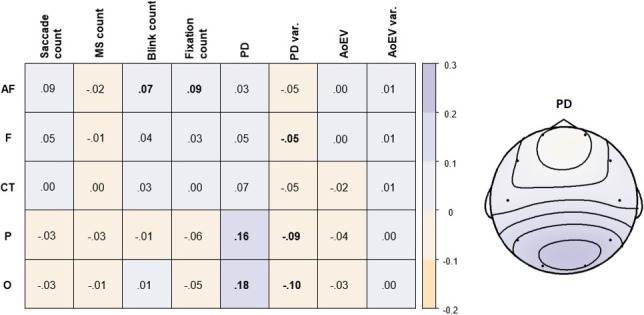
Correlations between electroencephalography (EEG) alpha power and eye parameters during the 120 s rest period (left). Bold numbers denote significant average correlations (*p* < .00125). Topographical plot of correlations between pupil diameter (PD) and EEG alpha power for aggregated channels (right). Violet tones denote positive correlations, and orange tones denote negative correlations. AoEV, angle of eye vergence; MS, microsaccade; var., variance

In a next step, we computed the Pearson correlation between the intrinsic variation of EEG alpha power and all available eye parameters during rest (Figure 4). EEG alpha power and PD were positively correlated over time, and their correlation was more pronounced toward posterior brain regions in both hemispheres (for scatter plots, see Figure C in the Supporting Information). This indicates that spontaneous increases of posterior EEG alpha power were related to concurrent increases in pupil diameter. EEG alpha power also showed small negative correlations with PD variance.

We further observed positive correlations of frontal EEG alpha power with blink count and fixation count. Blinks and eye movements are known to affect EEG alpha activity, especially at frontopolar sites (Hagemann & Naumann, 2001; Plöchl, Ossandón, & König, 2012). Control analyses showed that alpha power at EOG electrodes was also substantially correlated with AF alpha power (*r* ~ .89). In addition, alpha power at EOG electrodes and AF alpha power showed similar positive correlations with blink and fixation count (*r* ~ .10). Hence, despite approaching the removal of blink and saccade artifacts conservatively, it cannot be fully ruled out that the temporal correlations between frontal alpha power and the frequency of blinks and fixations are driven by a remaining effect of eye movements on the frontal EEG signal. It should be noted, however, that EEG alpha power at posterior brain regions was uncorrelated to blinks and fixations. EEG alpha power was not significantly correlated to the temporal variation of any other eye parameter (i.e., microsaccades, saccades, AoEV and AoEV variance) at the 0.00125 level (Bonferroni corrected).

## 4 DISCUSSION

This EEG—eye‐tracking coregistration study tested whether EEG alpha power and eye behavior are reliable indicators of internal focus of attention and explored their intrinsic temporal relationship. Using an established paradigm to realize within‐task manipulations of internal versus external attention demands in the visual domain, we replicated the finding that an internal focus of attention is associated with relatively higher EEG alpha power in posterior brain regions (i.e., lower desynchronization; Benedek, 2018; Fink & Benedek, 2013) as well as with specific eye behavior changes such as increased pupil diameter (PD) and reduced microsaccade rate (Benedek et al., 2017; Gao et al., 2015). We further found that EEG alpha activity and PD do not only show task‐induced attention effects but also covary over time during rest, which suggests that they both play a role in the modulation of perception and sustained IDC, potentially by being involved in a common neurophysiological gating mechanism.

This study realized an experimental manipulation of internal attention demands within tasks, similar to a previous EEG study (Benedek et al., 2011). Minor modifications to the original study included the use of a fixed trial duration (20 s) instead of self‐paced responding in order to increase consistency of task performance across trials and participants; this trial duration was found to provide sufficient time for task performance in the original study and resulted in similarly high solution rates in this study. Further differences included the use of active electrodes to increase signal‐to‐noise ratio, and the coregistration of eye behavior, which enabled a precise, direct detection of blink and saccadic activity enabling objective artifact correction. Despite these adaptations, this study clearly replicated the main findings from the original study. Again, higher internal processing demands resulted in relatively higher EEG alpha activity (i.e., lower desynchronization) especially in posterior brain regions (Benedek et al., 2011). Moreover, the same trend toward a moderation by task and hemisphere was observed suggesting relatively higher alpha power during IDC in right‐posterior brain regions specifically in the divergent thinking task. Our findings are consistent with other studies showing higher EEG alpha power in posterior brain regions for other forms of IDC (Agnoli et al., 2020; Cooper et al., 2003; Fink & Benedek, 2014; Ray & Cole, 1985; Stevens & Zabelina, 2019), suggesting that posterior alpha power is a robust indicator of internally directed attention. At the same time, it should be noted that we did not observe increases in alpha power as compared to a reference period, but instead found lower alpha desynchronization which still reflects relatively higher alpha power during IDC as compared to EDC. Future studies should investigate whether the observed relatively higher alpha power might be due to higher phase‐locking in response to stimulus onset rather than increased amplitudes of alpha oscillation (e.g., Rominger et al., 2020; Samaha, Bauer, Cimaroli, & Postle, 2015), as phase‐locking in the alpha band is also linked to visual WM encoding (Freunberger, Fellinger, Sauseng, Gruber, & Klimesch, 2009; Haenschel, Linden, Bittner, Singer, & Hanslmayr, 2010). Such investigations may inform deliberations on the specific functional roles of alpha activity (cf. Clayton, Yeung, & Cohen Kadosh, 2018; Jensen et al., 2012; Klimesch, 2012).

Since internal attention conditions required to keep the stimuli in mind, it rendered task performance slightly more difficult than external attention conditions as evidenced by lower solution rates and higher subjective task difficulty. This raises the question whether the observed alpha effects are merely due to increased task difficulty. We believe that this alternative explanation does not apply for the following two reasons: First, while we observed a strong task effect on task difficulty (SG was more difficult than AN), we observed no task effect on EEG alpha power. Second, previous work found that an easier divergent thinking task (higher solution rate) can still exhibit considerably higher alpha activity, especially when it is an intrinsically sensory‐independent task (Benedek et al., 2014). Together, this suggests that relatively higher EEG alpha activity is not just an effect of task difficulty but may rather resemble an effect of memory load. IDC tasks require to keep all relevant information in working memory, which typically implies increased memory load. In fact, posterior alpha activity has been shown to linearly increase with memory load in a short‐term memory task (Jensen et al., 2002). As memory load increases, attention needs to be focused internally more exclusively (Chatham & Badre, 2015), and alpha synchronization may serve the function of inhibition of task‐irrelevant areas (Jensen & Mazaheri, 2010). Hence, posterior alpha activity during IDC is assumed to represent functional inhibition of visual processing to shield ongoing internal processing (Benedek, 2018; Jensen et al., 2012; Klimesch, 2012).

This study also replicated most findings of a previous eye‐tracking study (Benedek et al., 2017). As in the original study, IDC compared to EDC was associated with lower fixation and saccade count, higher fixation duration, higher saccade amplitude, lower microsaccade count, and larger PD. The successful replication highlights the robustness of these findings. Unlike the original study, this study did not find significant effects on PD variance, blink duration, AoEV, or AoEV variance. Notably, the interpretation of AoEV findings may be limited by the accuracy of eye trackers (Hooge, Hessels, & Nyström, 2019). Pupil diameter increases during IDC appear to be a rather consistent finding in the literature (Annerer‐Walcher, Körner, & Benedek, 2018; Benedek et al., 2017; Franklin et al., 2013; Smallwood et al., 2012; Walcher et al., 2017), although some studies revealed opposing trends (Unsworth & Robinson, 2016; Grandchamp et al., 2014). The pupil diameter is known to reflect to changes in workload (Beatty, 1982; Kahneman & Beatty, 1966), which is typically increased during many goal‐directed forms of IDC. On the other hand, PD increases are also linked to orientation of attention toward external events (Sara & Bouret, 2012). These findings suggest that increases of PD are plausible during both IDC and EDC, and factors such as task characteristics, task focus, and time‐on‐task effects (e.g., van den Brink, Murphy, & Nieuwenhuis, 2016) need to be weighed in when interpreting PD in the context of visual attention.

Other eye behavior differences between IDC and EDC suggest that IDC implies a state of visual disengagement. Although neither blink rate nor blink duration significantly increased during IDC (after Bonferroni correction), our results do indicate a tendency toward longer periods of shutting down visual inflow in line with other studies (blink rate: Smilek, Carriere, & Cheyne, 2010; Grandchamp et al., 2014; Walcher et al., 2017; Annerer‐Walcher et al., 2018; blink duration: Walcher et al., 2017; Benedek et al., 2017), which has been linked to attention and information processing (Liu, Hajra, Cheung, Song, & Arcy, 2017). Moreover, microsaccade count decreased during IDC, which is consistent with previous findings (Benedek et al., 2017; Walcher et al., 2017). Since microsaccades serve as a mechanism to maintain stable vision while fixating an object by avoiding perceptual habituation (Duchowski, 2017; Martinez‐Conde, Macknik, & Hubel, 2004), a reduction in microsaccadic activity during IDC implies a fading of (irrelevant) visual input (McCamy et al., 2012). Together, lower microsaccade rate and a slight trend toward increased blink duration represent a pattern of reduced preparedness to detect and process visual information, which evidences a state of visual disengagement. Active perceptual suppression of task‐irrelevant sensory information by means of eye closure or gaze aversion was in fact shown to be characteristic for moments of spontaneous “insight” (Salvi et al., 2015) and to facilitate performance across different IDC tasks (Doherty‐Sneddon & Phelps, 2005; Ritter et al., 2018).

We also replicated that fixation and saccade rates decrease during IDC and that saccade amplitude increases when attention was directed internally. The interpretation of differences in saccade rates requires to consider task characteristics: As the AN and SG tasks involved the processing of 4‐letter‐stimuli, participants likely made more saccadic movements between the letters when they were meaningful (EDC condition). Yet, the small stimulus at the center of the screen also confined eye movements, resulting in generally lower saccadic amplitude during EDC. Similar to saccades, findings regarding fixations match previous results (Benedek et al., 2017). They are also in line with research regarding mindless reading, suggesting that during IDC, we process letters less systematically, resulting in fewer, yet longer fixations (Reichle et al., 2010). Similarly, the small trend toward an increase in PD variance during internal attention is in line with literature regarding perceptual decoupling, showing that eye behavior becomes less determined by the visual environment and thus more spontaneous during IDC (Smallwood et al., 2011). In sum, eye behavior changes during IDC reflect a characteristic pattern of increased memory load, reduced visual engagement, and perceptual decoupling. Future studies should investigate whether shifting from nonvisual EDC (e.g., auditory information) to IDC evokes eye behavior changes similar to manipulations of visual attention as in this study.

### 4.1 Covariation between EEG alpha activity and eye parameters

As a central novelty of this study, we examined whether intrinsic variation in EEG alpha power is associated with relevant eye behavior changes. This analysis was applied to resting state data, where attentional focus is not determined by a given task and thus reflects spontaneous, transient changes in the focus of attention between the environment and inner processes (Smallwood & Schooler, 2015), and may indicate fluctuations of vigilance (Langner & Eickhoff, 2013). We found that EEG alpha activity was positively correlated with PD across time. Correlations were most pronounced in posterior brain regions, which is just where EEG alpha showed the strongest attention effects. Moreover, this finding also fits the observation that PD showed the strongest task‐based attention effects of all eye parameters. Considering that experimentally induced internal attention resulted in relatively higher posterior EEG alpha power and PD (see also Figure D in the Supporting Information), the temporal correlation during rest suggests that EEG alpha and PD represent time‐critical indicators of an internal versus external attentional demands. As EEG alpha activity is associated with top‐down inhibition of task‐irrelevant brain areas and PD is, among other things, linked to memory load, this correlation further suggests that EEG alpha and PD might be involved in the modulation of perception and the shielding of ongoing internal processes especially in face of higher internal attention demands. Such input gating to working memory may further involve the basal ganglia in the context of the cortico‐basal ganglia circuit (Chatham & Badre, 2015). Future research should aim to directly assess spontaneous shifts of attention during resting state, which could be done by means of attention probes (Smallwood & Schooler, 2015) or by means of independent physiological assessments (e.g., Annerer‐Walcher et al., in press).

It is also an important to note that EEG alpha activity can serve different functions in sustained attention and thus may play different roles in resting state activity compared to task‐related activity (Clayton et al., 2018). Our findings, indicating that alpha activity and PD covary during rest as well as in response to a given task, suggest a link between resting state alpha and task‐related alpha via its association with PD. While spontaneous shifts of attention to internal events are commonly associated with mind wandering and thus low task focus, the tasks in this study required internal attention but high task focus. Hence, while our findings may speak to the role of alpha activity and PD in internal attention, they may not easily generalize to related concepts of task focus or vigilance.

Interestingly, we also observed a small negative correlation between EEG alpha power and PD variance, although PD variance was tendentially increased during task‐related IDC. This finding could be partly attributed to the fact that PD variance computation referred to a much smaller time window for temporal covariation analyses compared to task‐based analyses (1 s vs. 18.5 s) and may point to potentially different validity of this measure for spontaneous versus goal‐directed forms of IDC. We also explored temporal correlations between EEG alpha power and discrete eye parameters (i.e., saccades, microsaccades, blinks, and fixations). These analyses yielded no significant associations with EEG alpha power, except for blinks and fixations in frontal areas. While these associations might reflect relevant brain activity such as activation of frontal eye fields (Schall, 2004), these results need to be interpreted very cautiously, as they may also result from well‐known eye movement artifacts on the EEG signal. Investigating the relationship of EEG alpha power and discrete eye events appears to be challenged by their unfavorable temporal characteristics (i.e., rare appearance) and their known effect on the EEG signal (e.g., blinks). Despite a conservative approach in regard to artifact removal, we cannot fully rule out that some effects of eye movements on the EEG signal may still affect the brain activation measurements at frontal regions. Yet, our analyses also indicate that alpha power assessments in posterior brain regions, where the main attention effects were observed in this and previous studies, appear largely unaffected by blink artifacts.

### 4.2 Summary and conclusion

This study provided additional evidence that IDC is consistently associated with relatively higher EEG alpha activity over posterior brain regions, which is viewed to serve the inhibition of task‐irrelevant sensory processing. IDC was further associated with specific eye behavior changes that reflect a pattern of visual disengagement (i.e., fewer microsaccades and tendentially longer blinks), perceptual decoupling (i.e., fewer fixations/saccades and a trend toward increased PD variance), and increased memory load (i.e., higher PD). Importantly, we found that EEG alpha activity and PD do not only show a consistent response to increased internal attention demands but are also positively correlated over time during rest. Together, these findings suggest that posterior alpha activity and PD represent time‐sensitive indicators of internal attention demands, which may be involved in a neurophysiological gating mechanism serving to shield internal cognition from irrelevant sensory information.

## CONFLICT OF INTEREST

The authors declare no conflict of interest.

## AUTHOR CONTRIBUTION

S.M.C. collected the data. S.M.C. and S.A.‐W. prepared the analysis scripts. S.M.C. performed analyses. S.M.C., S.A.‐W., C.K., C.R., S.E.K., A.F., and M.B. wrote the manuscript. S.A.‐W., C.K., and M.B. conceived the study and were in charge of overall direction and planning.

## ETHICS APPROVAL STATEMENT

This study was approved by the local ethics committee.

### Peer Review

The peer review history for this article is available at https://publons.com/publon/10.1002/brb3.1790.

## Supporting information

Supplementary Material

## Data Availability

Materials, data, and analysis scripts are provided on the Open Science Framework (OSF, https://doi.org/10.17605/OSF.IO/5U6R9).
