# Supplementary material for: Neurophysiological indicators of internal attention: An electroencephalography–eye‐tracking coregistration study
Source: Brain Behav. 2020 Aug 20;10(10):e01790. doi: 10.1002/brb3.1790 (PMC7559625; doi:10.1002/brb3.1790)
Supplement: Supplementary file 1 — Supplementary Material [file BRB3-10-e01790-s001.docx]

**Supplementary material:** Neurophysiological indicators of internal attention: An EEG-eye-tracking co-registration study

| Table 1. |  |  |  |  |  |  |  |  |  |  |  |  |  |  |  |
| --- | --- | --- | --- | --- | --- | --- | --- | --- | --- | --- | --- | --- | --- | --- | --- |
| *Mean TRP (and SE) for each area during externally directed cognition (EDC) and internally directed cognition (IDC) for each task and hemisphere.* | | | | | | | | | | | |  |  |  |  |
|  |  |  |  |  |  | **EDC** |  |  |  |  |  |  |  |  |  |
|  |  | **AN** |  |  |  | **SG** |  |  |  | **total** |  |  |  |  |  |
|  | left | right | total |  | left | right | total |  | left | right | total |  |  |  |  |
| AF | 0.46 (0.12) | 0.48 (0.12) | 0.47 (0.08) |  | 0.59 (0.13) | 0.63 (0.14) | 0.61 (0.09) |  | 0.52 (0.09) | 0.56 (0.09) | 0.52 (0.06) |  |  |  |  |
| F | 0.02 (0.05) | 0.02 (0.06) | 0.02 (0.04) |  | 0.03 (0.06) | 0.01 (0.06) | 0.02 (0.04) |  | 0.02 (0.04) | 0.01 (0.04) | 0.02 (0.03) |  |  |  |  |
| CT | -0.16 (0.04) | -0.08 (0.04) | -0.12 (0.03) |  | -0.17 (0.05) | -0.13 (0.03) | -0.15 (0.03) |  | -0.16 (0.03) | -0.10 (0.03) | -0.13 (0.02) |  |  |  |  |
| P | -0.33 (0.07) | -0.32 (0.06) | -0.33 (0.04) |  | -0.33 (0.06) | -0.32 (0.05) | -0.33 (0.04) |  | -0.33 (0.04) | -0.32 (0.04) | -0.33 (0.03) |  |  |  |  |
| O | -0.37 (0.07) | -0.30 (0.05) | -0.33 (0.04) |  | -0.34 (0.07) | -0.32 (0.05) | -0.33 (0.04) |  | -0.35 (0.05) | -0.31 (0.04) | -0.33 (0.03) |  |  |  |  |
|  |  |  |  |  |  |  |  |  |  |  |  |  |  |  |  |
|  |  |  |  |  |  | **IDC** |  |  |  |  |  |  |  |  |  |
|  |  | **AN** |  |  |  | **SG** |  |  |  | **total** |  |  |  |  |  |
|  | left | right | total |  | left | right | total |  | left | right | total |  |  |  |  |
| AF | 0.47 (0.09) | 0.53 (0.09) | 0.50 (0.07) |  | 0.62 (0.12) | 0.67 (0.13) | 0.64 (0.09) |  | 0.54 (0.08) | 0.60 (0.08) | 0.57 (0.05) |  |  |  |  |
| F | 0.08 (0.04) | 0.09 (0.04) | 0.09 (0.03) |  | 0.08 (0.05) | 0.11 (0.06) | 0.09 (0.04) |  | 0.08 (0.03) | 0.10 (0.04) | 0.09 (0.02) |  |  |  |  |
| CT | -0.07 (0.04) | -0.04 (0.03) | -0.05 (0.02) |  | -0.11 (0.03) | -0.08 (0.03) | -0.10 (0.02) |  | -0.09 (0.02) | -0.06 (0.02) | -0.08 (0.02) |  |  |  |  |
| P | -0.17 (0.06) | -0.17 (0.06) | -0.17 (0.04) |  | -0.24 (0.05) | -0.17 (0.04) | -0.20 (0.03) |  | -0.21 (0.04) | -0.17 (0.04) | -0.19 (0.03) |  |  |  |  |
| O | -0.13 (0.05) | -0.12 (0.05) | -0.12 (0.04) |  | -0.23 (0.07) | -0.11 (0.05) | -0.17 (0.04) |  | -0.18 (0.04) | -0.11 (0.03) | -0.15 (0.03) |  |  |  |  |
| *Note. AF = anteriofrontal, F = frontal, CT = centro-temporal, P = parietal, O = occipital; AN = anagram generation, SG = sentence generation; left = left hemisphere, right = right hemisphere* | | | | | | | | | | | |  |  |  |  |

| Table 2. |  |  |  |  |  |  |  |  |
| --- | --- | --- | --- | --- | --- | --- | --- | --- |
| *Mean (and SE) for each ocular parameter during externally directed cognition (EDC) versus internally directed cognition (IDC) for both tasks.* | | | | | | | | |
|  |  |  | **EDC** |  |  |  | **IDC** |  |
|  |  | **AN** | **SG** | **Total** |  | **AN** | **SG** | **Total** |
| Fixation count [1/s] | | 1.07 (0.08) | 1.03 (0.05) | 1.05 (0.05) |  | 0.78 (0.06) | 0.86 (0.06) | 0.82 (0.04) |
| Fixation duration [ms] | | 119.08 (26.84) | 87.54 (9.97) | 103.31 (14.34) |  | 144.17 (27.11) | 105.69 (10.00) | 124.93 (14.53) |
| Saccade count [1/s] | | 0.79 (0.08) | 0.68 (0.05) | 0.74 (0.05) |  | 0.48 (0.06) | 0.52 (0.06) | 0.50 (0.04) |
| Saccade amplitude [degree] | | 1.29 (0.02) | 1.34 (0.02) | 1.32 (0.01) |  | 1.79 (0.19) | 1.63 (0.14) | 1.71 (0.12) |
| Microsaccade count [1/s] | | 1.42 (0.12) | 1.33 (0.11) | 1.38 (0.08) |  | 0.88 (0.10) | 1.00 (0.12) | 0.94 (0.08) |
| Microsaccade amplitude [degree] | | 0.51 (0.01) | 0.50 (0.02) | 0.50 (0.01) |  | 0.46 (0.02) | 0.48 (0.02) | 0.47 (0.01) |
| Blink count [1/s] | | 0.31 (0.04) | 0.37 (0.05) | 0.34 (0.03) |  | 0.35 (0.04) | 0.39 (0.05) | 0.37 (0.03) |
| Blink duration [ms] | | 90.93 (5.70) | 87.53 (4.42) | 89.23 (3.59) |  | 116.12 (8.55) | 108.82 (16.98) | 112.47 (9.45) |
| Pupil diameter | | -0.01 (0.05) | 0.53 (0.05) | 0.26 (0.05) |  | 0.21 (0.06) | 0.77 (0.04) | 0.49 (0.05) |
| Pupil diameter variance | | 0.14 (0.01) | 0.17 (0.02) | 0.16 (0.01) |  | 0.19 (0.02) | 0.18 (0.02) | 0.18 (0.01) |
| AoEV [degree] | | 5.33 (0.14) | 5.27 (0.14) | 5.30 (0.10) |  | 5.30 (0.13) | 5.22 (0.15) | 5.26 (0.10) |
| AoEV variance [degree²] | | 0.06 (0.01) | 0.07 (0.01) | 0.06 (0.01) |  | 0.08 (0.01) | 0.07 (0.01) | 0.08 (0.01) |
| *AN = anagram task; SG = sentence generation task; AoEV = angle of eye vergence.* | | | | | | | | |

Figure A. Effects of blinks (as classified by the eye tracker) on EEG alpha power. Graphs show an aggregation of blink data during task performance (blue lines) from three electrode positions (Fp1, C3 and O1) +/- SE of all subjects for blink start (top) and blink end (bottom). Vertical lines mark avg. blink classification by the eye tracker (red) and corrected classifications of blink on/offsets after visual inspection (black).


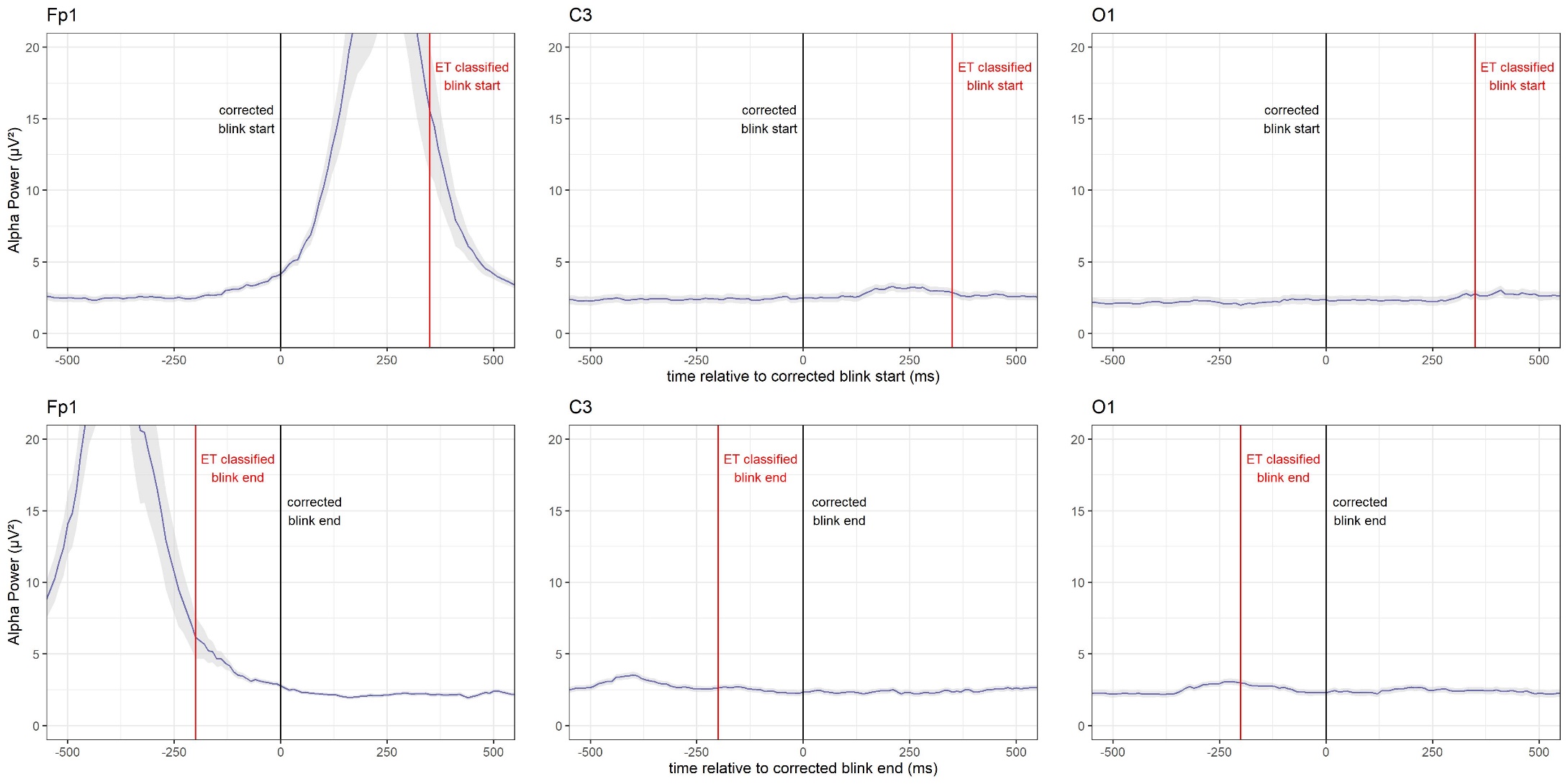


*Figure B*. Average alpha power for the resting-state and task-based EEG. Dashed vertical lines denote the 8.5-12.5 Hz alpha window used in this study. EEG channel Pz is shown.


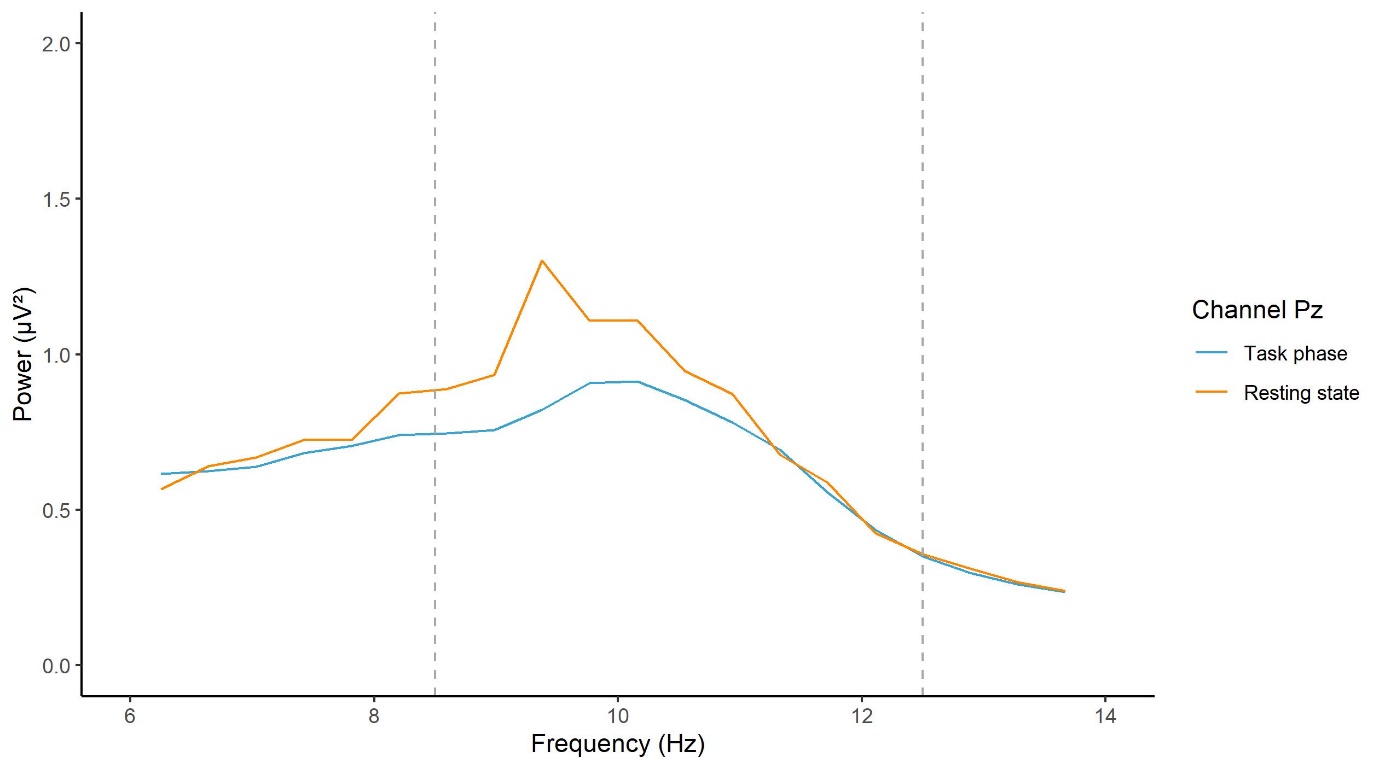

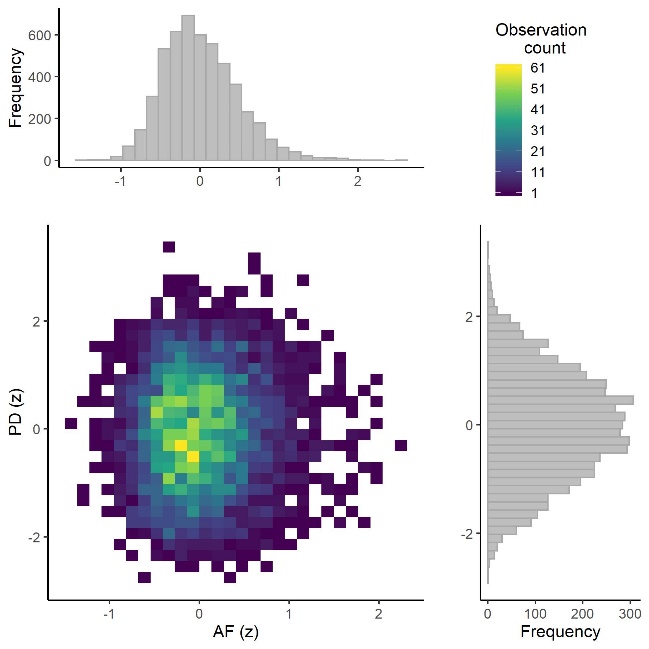

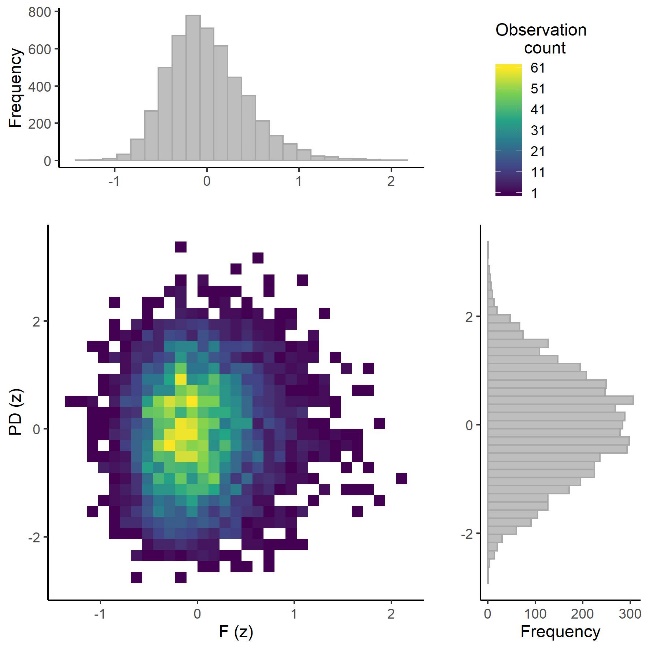

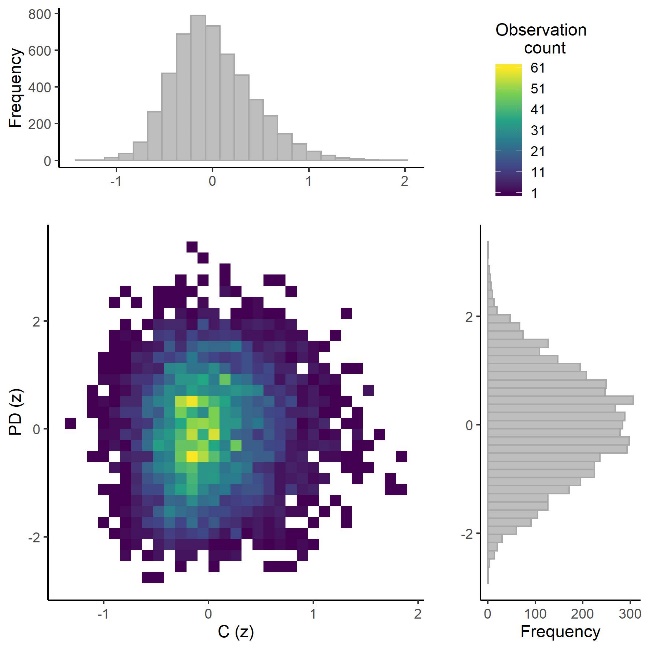

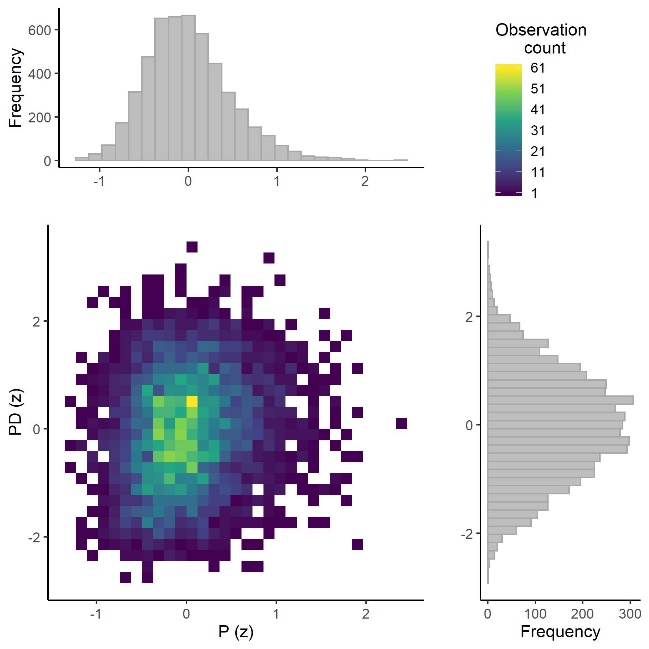

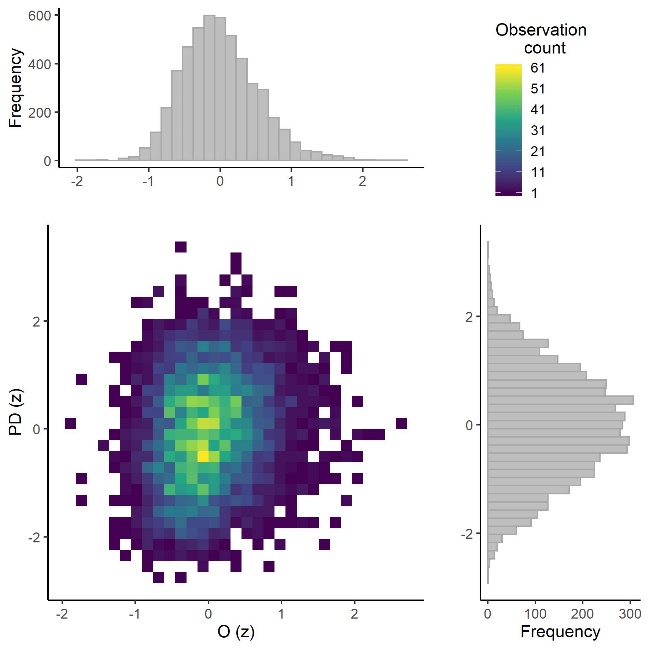


*Figure C.* Relationship between PD and aggregated EEG channels (both z-scored). Top left = AF; top right = F; center left = C; center right = P; bottom left = O. Histograms show distribution of PD (right side) and aggregated EEG channels (top side). Brighter spots in the main graphs denote higher observation count.


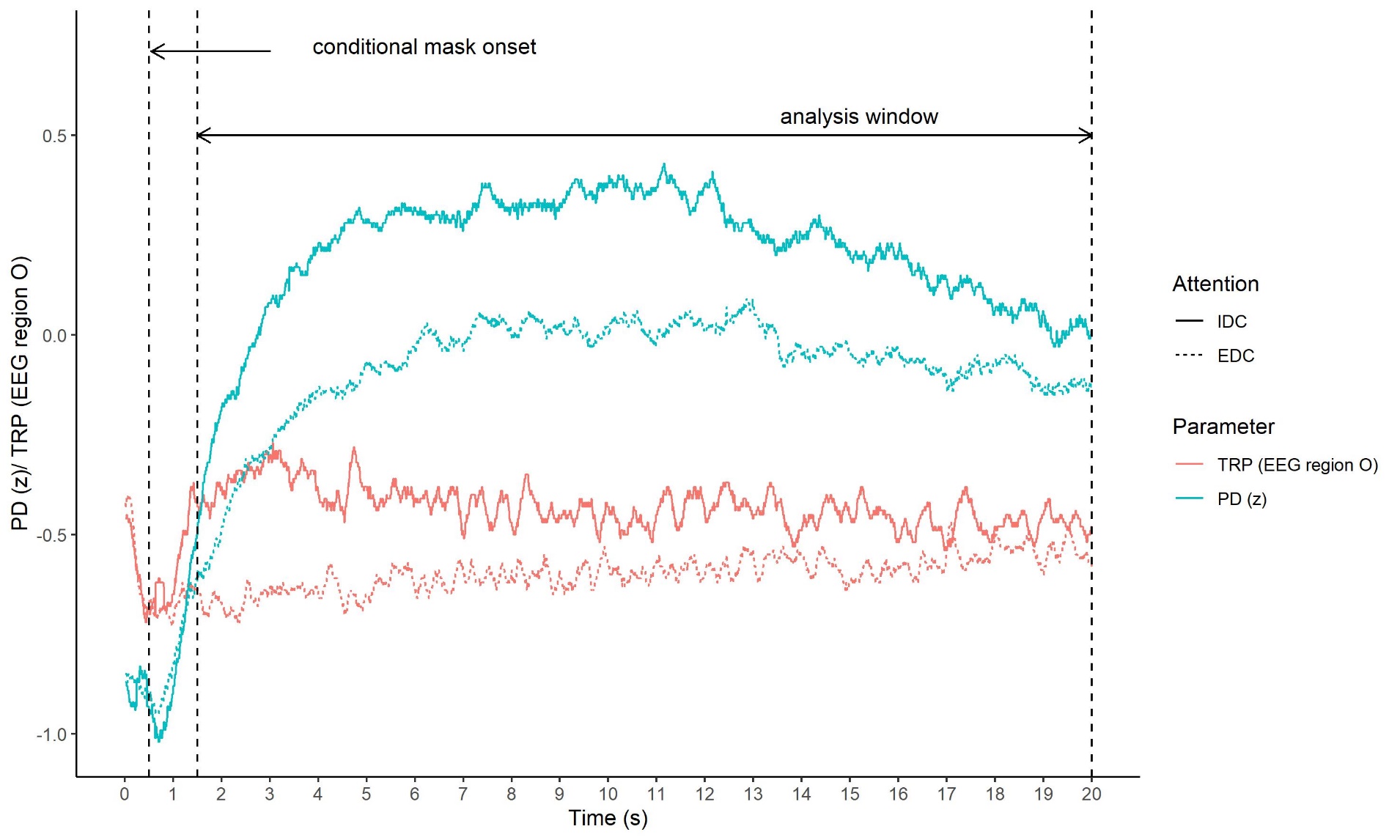


*Figure D.* Time-course of average alpha activity (task-related power for the aggregated occipital region) and average PD (z-scored) along the 20s trial window for IDC (solid) and EDC (dashed), respectively. Vertical lines mark conditional mask onset (after 500ms) and start/end of analysis window (1.5s/20s, respectively).
